# Urobiota analysis and genome-wide association study in pediatric recurrent urinary tract infections and vesicoureteral reflux

**DOI:** 10.1172/jci.insight.199689

**Published:** 2025-12-09

**Authors:** Miguel Verbitsky, Pavan Khosla, Daniel Bivona, Atlas Khan, Yask Gupta, Heekuk Park, Tian H. Shen, Aryan Ghotra, Katherine Xu, Iman A. Ghavami, Priya Krithivasan, Jeremiah Martino, Tanya Sezin, Tze Y. Lim, Victoria Kolupaeva, Nita A. Limdi, Yuan Luo, Hakon Hakonarson, Simone Sanna-Cherchi, Krzysztof Kiryluk, Cathy L. Mendelsohn, Anne-Catrin Uhlemann, Jonathan Barasch, Ali G. Gharavi

**Affiliations:** 1 Department of Medicine, Division of Nephrology;; 2 Department of Medicine, Division of Infectious Diseases;; 3 Department of Dermatology; and; 4 Center for Precision Medicine and Genomics, Columbia University, New York, New York, USA.; 5 Department of Neurology, Heersink School of Medicine, University of Alabama at Birmingham, Birmingham, Alabama, USA.; 6 Division of Statistics and Informatics, Northwestern University Feinberg School of Medicine, Chicago, Illinois, USA.; 7 Center for Applied Genomics, The Children’s Hospital of Philadelphia and Perelman School of Medicine at the University of Pennsylvania, Philadelphia, Pennsylvania, USA.; 8 Department of Urology, Columbia University, New York, New York, USA.

**Keywords:** Genetics, Infectious disease, Nephrology, Bacterial infections, Genetic risk factors, Microbiome

## Abstract

Urinary tract infections (UTIs) are the most common severe bacterial infections in young children, often associated with vesicoureteral reflux (VUR). To explore host genetic-microbiota interactions and their clinical implications, we analyzed the urinary microbiota (urobiota) and conducted genome-wide association studies for bacterial abundance traits in pediatric patients with UTI and VUR from the Randomized Intervention for Children with Vesicoureteral Reflux and Careful Urinary Tract Infection Evaluation cohorts. We identified 4 urobiota community types based on relative abundance, characterized by the genera *Enterococcus*, *Prevotella*, *Pseudomonas*, and *Escherichia/Shigella*, and their associations with VUR, age, and toilet training. Children with VUR exhibited decreased microbial diversity and increased abundance of genera that included opportunistic pathogens, suggesting a disrupted urobiota. We detected genome-wide significant genetic associations with urinary bacterial relative abundances, in or near candidate genes including *CXCL12*, *ABCC1*, and *ROBO1*, which are implicated in urinary tract development and response to infection. We showed that *Cxcl12* was induced 12 hours after uropathogenic bacterial infection in mouse bladder. The association with *CXCL12* suggests a genetic link between UTI, VUR, and cardiovascular phenotypes later in life. These findings provide the first characterization to our knowledge of host genetic influences on the pediatric urobiota in UTI and VUR, offering insights into the interplay between disease, host genetics, and the urobiota composition.

## Abstract 2

Genetic variations influence bacterial composition of urine in children with recurrent urinary infections and vesicoureteral reflux.

## Introduction

Urinary tract infections (UTIs) are the most frequent severe bacterial infections in young children (1). Up to 8.4% of girls and 1.7% of boys will have a UTI in the first 6 years of life (2). Vesicoureteral reflux (VUR), which allows the flow of urine from the bladder back to the kidneys, is present in one-third of children presenting with febrile UTI and is linked to increased risk of renal scarring (3). Bladder and bowel dysfunction may also be significant contributors to the recurrence of UTI. In bacterial cultures from pediatric UTIs, *Escherichia coli*, *Klebsiella*, *Enterococcus*, *Proteus*, and *Pseudomonas* spp are most commonly found. *Pseudomonas* spp, in particular, are more frequent in children undergoing antibiotic prophylaxis treatment and those with urinary tract anomalies (4–8). Symbiotic bacterial communities predominantly constitute the bacterial microbiota in the asymptomatic, presumably healthy urinary tract. In contrast, urinary dysbiosis has been implicated in pathologic states such as nephrolithiasis, benign prostatic hyperplasia, bladder cancer, urinary incontinence, and recurrent infections and reported in kidney transplant patients (9–12).

We hypothesized that host genetic factors predispose to variation in urinary microbiota and surveyed host genetic-microbiota associations in pediatric patients with UTI, with or without VUR, in whom they could have a significant and lasting impact. We performed our study in participants in 2 studies sponsored by the NIH National Institute of Diabetes and Digestive and Kidney Diseases (NIDDK), the Randomized Intervention for Children with Vesicoureteral Reflux (RIVUR) study, which recruited children with both UTI and VUR, and the Careful Urinary Tract Infection Evaluation (CUTIE) study, a RIVUR ancillary study whose participants were initially evaluated for inclusion in RIVUR and excluded because of the absence of VUR (2, 13, 14).

## Results

### Microbiota analysis.

**Figure 1:**
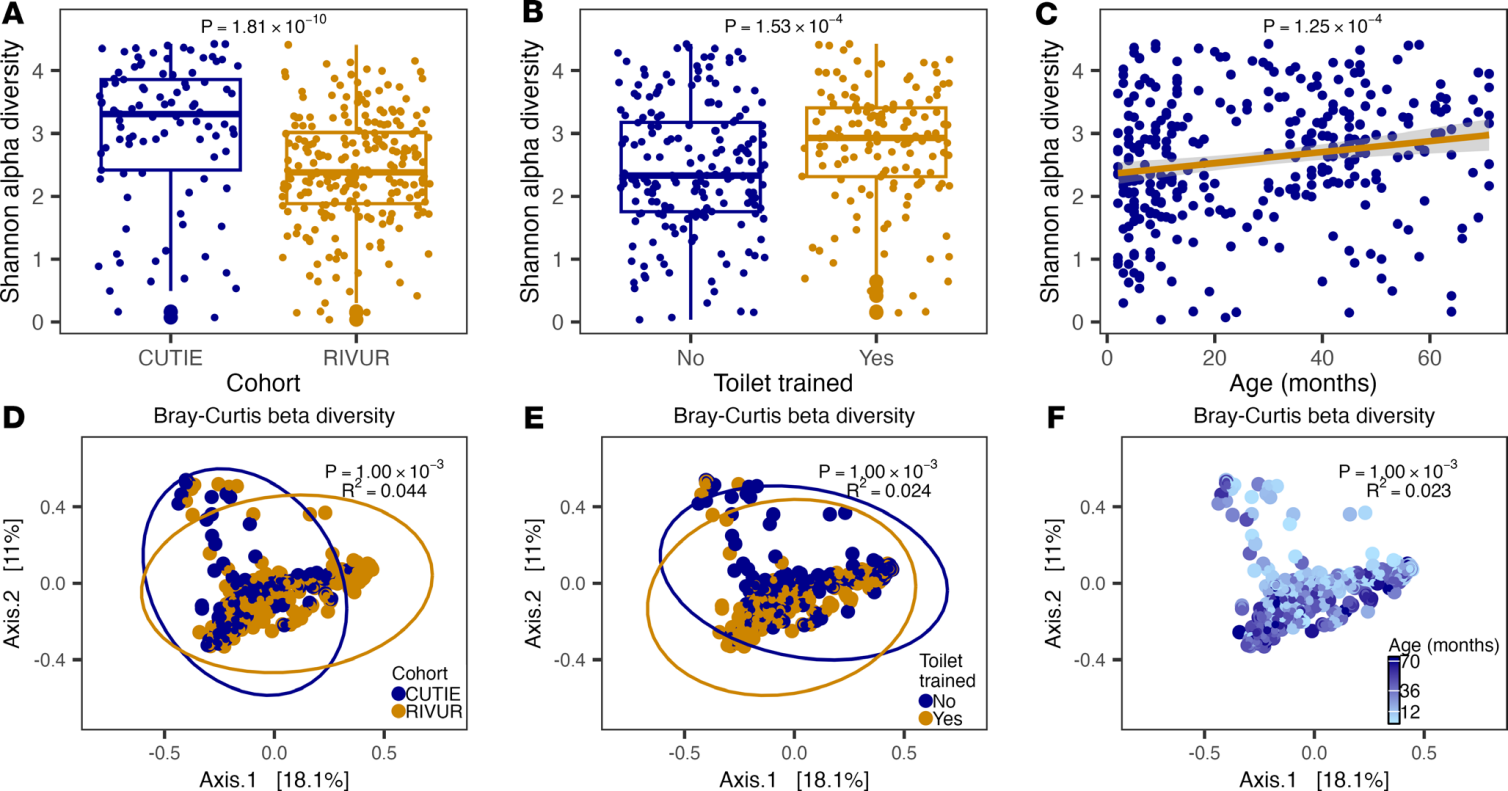
Urobiota α- and β-diversity associations. Shannon α-diversity (top) and Bray-Curtis β-diversity (bottom) were computed and tested for differences between RIVUR and CUTIE cohorts (**A** and **D**), with toilet training (**B** and **E**), and age (**C** and **F**). Rank-based regression was used for α-diversity and PERMANOVA for β-diversity. In the α-diversity plots the bottom, middle, and top horizontal lines of the box represent the 25th, 50th, and 75th percentiles, respectively; the bottom and top whiskers extend to the lowest and highest value within 1.5× IQR, respectively.

We performed 16S ribosomal RNA (rRNA) gene V3-V4 region sequencing of 325 urine samples from RIVUR (*n* = 228) and CUTIE (*n* = 97) (Supplemental Table 1; supplemental material available online with this article; https://doi.org/10.1172/jci.insight.199689DS1). We calculated per-sample Shannon α-diversity, a measure of species richness, evenness, or diversity within a sample, and between-sample β-diversity Bray-Curtis pairwise dissimilarity indices (BCd), a measure of dissimilarity between 2 or more communities. We observed a significant decrease in Shannon α-diversity, with VUR (*P* = 1.81 × 10^–10^; *P* = 2.29 × 10^–8^, when correcting for race, ethnicity, and age), lack of toilet training (*P* = 1.53 × 10^–4^), younger age (*P* = 1.25 × 10^–4^), and higher systolic (*P* = 1.77 × 10^–3^) and diastolic (*P* = 2.58 × 10^–2^) blood pressure (Figure 1, Supplemental Methods, and Supplemental Figure 1). Similarly, principal coordinate analysis and permutational ANOVA on the BCd matrix (Adonis test, *n* permutations = 999) showed significant differences with cohort (*R*^2^ = 0.044, *P* = 0.001; *R*^2^ = 0.031, *P* = 0.001, when correcting for race, ethnicity, and age), toilet training (*R*^2^ = 0.024, *P* = 0.001), age (*R*^2^ = 0.023, *P* = 0.001), and systolic (*R*^2^ = 0.010, *P* = 0.021) and diastolic (*R*^2^ = 0.018, *P* = 0.001) blood pressure. We found that the number of UTI or antibiotic treatments showed no significant α-diversity (*P* > 0.05) association and explained less than 1% of variance in the BCd matrix (*R*^2^ = 0.006, *P* = 0.029 and *R*^2^ = 0.005, *P* = 0.039, respectively).

**Figure 2:**
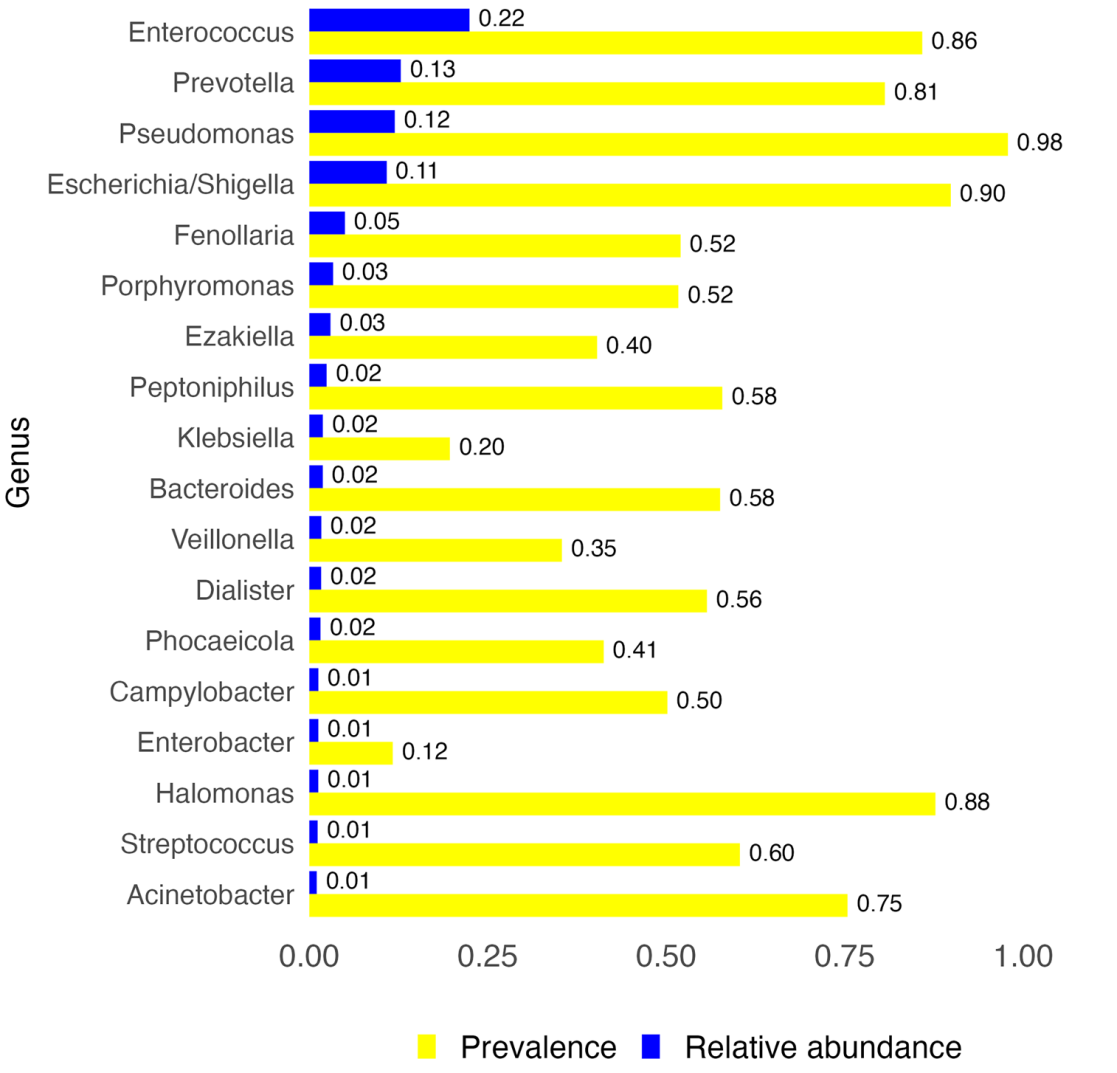
Urobiota composition, top genera. Aggregate relative abundance (blue bars) and prevalence (yellow bars) of top genera (relative abundance ≥ 0.01) in the urobiota of RIVUR and CUTIE participants (*N* = 325). Aggregate relative abundance and prevalence values are also shown as numbers at the end of each bar.

Overall, the most abundant genera were *Enterococcus*, *Prevotella*, *Pseudomonas*, and *Escherichia/Shigella* (0.22, 0.13, 0.12, and 0.11 aggregate relative abundances, respectively), which were also among the most prevalent (>80%, Figure 2 and Supplemental Figure 2).

Clustering of the samples based on Jensen-Shannon divergence (JSD) distance metrics of the microbiota at the genus level identified 4 community types (clusters) where the most abundant genera were *Pseudomonas*, *Prevotella*, *Escherichia/Shigella*, and *Enterococcus*, respectively (Supplemental Figure 3). We found associations of these community types with cohort, sex, age, toilet training, number of UTIs, and presence of scarring or pyelonephritis, at baseline (global Fisher’s exact and Kruskal-Wallis tests *P* = 1.02 × 10^–7^, 1.45 × 10^–4^, 4.47 × 10^–6^, 8.66 × 10^–7^, 2.10 × 10^–2^, and 2.52 ×10^–2^, respectively; Supplemental Tables 2 and 3). Specifically, in 2-cluster comparisons, we found that the Pseudomonas and Enterococcus clusters were enriched compared with each of the other clusters in VUR (RIVUR cohort). The Prevotella cluster was enriched in females compared with the Pseudomonas cluster, with older age compared with the other clusters, with toilet training compared with the Pseudomonas and Enterococcus clusters, and with the number of UTIs and presence of scarring or pyelonephritis compared with the Pseudomonas cluster (Supplemental Tables 2 and 3).

**Figure 3:**
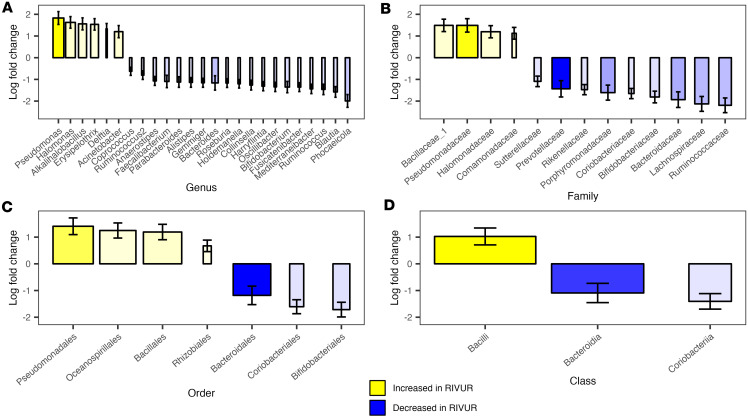
Differential bacterial taxa abundances between RIVUR and CUTIE cohorts. Bar plots represent analysis of compositions of microbiomes with bias correction (ANCOM-BC2) at the genus (**A**), family (**B**), order (**C**), and class (**D**) taxonomic levels. Increased abundance in RIVUR compared with CUTIE is represented in yellow and decreased abundance in blue. Color intensity is proportional to total relative abundance and bar width to prevalence across both cohorts. Only statistically significant (*q* < 0.05) differences associated with cohort (adjusted for age and sex) that could also be detected with MaAsLin2 are shown. Bar heights represent log fold-change and error bars their standard error (se).

Next, we tested for differential relative abundance of taxa in the core microbiota (defined as those bacterial taxa with a relative abundance greater than 0.001 in at least 10% of samples, at each taxonomic level), with a consensus approach based on 2 methods, ANCOM-BC2 (15) and MaAsLin2 (16) (Figure 3 and Supplemental Figures 4 and 5). Among the most abundant taxa (relative abundance > 0.1), genera *Pseudomonas* and *Halomonas*, family Pseudomonadaceae, order Pseudomonadales, and class Bacilli were increased in RIVUR relative to CUTIE (cohort, adjusted for sex and age; *q* < 0.05); families Prevotellaceae, Ruminococcaceae, and Lachnospiraceae, and class Bacteroidia, were decreased. Genus *Prevotella*, families Prevotellaceae and Peptoniphilaceae, orders Bacteroidales and Clostridiales, and classes Bacteroidia and Clostridia were increased, and family Enterobacteriaceae, order Enterobacterales, and class Gammaproteobacteria decreased with age and with toilet training (adjusted for sex and cohort; *q* < 0.05).

### Microbiota GWAS.

**Figure 4:**
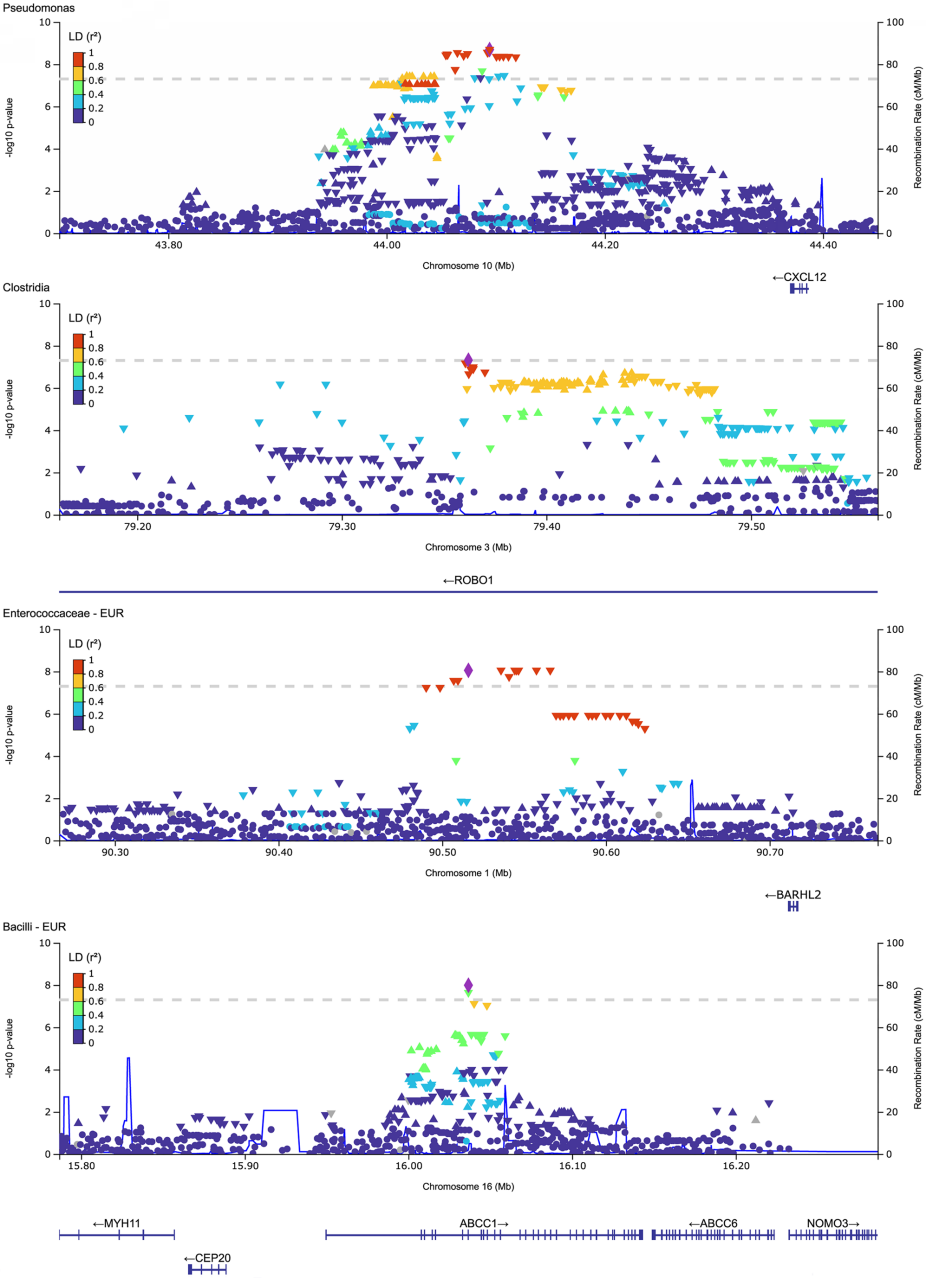
LocusZoom plots of top loci associated with urinary bacterial traits. Each row represents a different trait. REGENIE results are shown for the Pseudomonas and the Clostridia GWAS, and TRACTOR results on the EUR background, for the Enterococcaceae and the Bacilli GWAS. The *y* axes represent –log(*P* value) and the *x* axes, chromosomal coordinates (Mbp, hg38). SNP colors indicate linkage disequilibrium (LD) estimate (*r*^2^) ranges and the blue line recombination rate in centimorgans per megabase (cM/Mb). *CXCL12*, CXC motif chemokine ligand 12, also known as stromal cell-derived factor, *SDF1*; *ROBO1*, roundabout guidance receptor 1; *ABCC1*, ATP binding cassette-family transporter multidrug resistance protein 1 gene; *BARHL2*, BarH-like homeobox 2 gene.

We conducted whole-genome scans for associations of host genetic loci with urinary bacterial taxa relative abundances in a subset of 278 participants (Supplemental Table 4) with high-quality host genome genotyping data. Only samples with nonzero relative abundances were included in the analysis for these quantitative traits (17). We focused these analyses on 62 taxa detected in at least 50% of samples (Supplemental Figure 6). We first used the mixed effects regression model implemented in the REGENIE software (18). Since the study cohort is multiethnic, including individuals with admixed genetic ancestry, we also conducted tests for associations using TRACTOR, which implements a local ancestry-aware, fixed effects regression model, producing local ancestry-specific effect size estimates and *P* values (19). Genomic inflation factors ranged from 0.86 to 0.93 with REGENIE and 1.02 to 1.08 with TRACTOR. We prioritized genome-wide significant associations (*P* < 5 × 10^–8^) detected with at least one method and supported as suggestive (*P* < 10^–5^) by the other. Together, we identified 4 genome-wide significant associations (Table 1 and Figure 4), with no additional independent SNPs within these loci.

The strongest association was on chromosome 10 (chr 10), in an intergenic region downstream of *CXCL12*, with the relative abundances of genus *Pseudomonas* (top SNP: rs2624692, *P* = 2.03 × 10^–9^), and with that of the Pseudomonadaceae family (top SNP: rs2818904, *P* = 2.41 × 10^–8^). These lead SNPs on chr 10 are not independent (*r*^2^ = 0.93). For both SNPs, the major alleles were associated with lower *Pseudomonas* relative abundance in urine (Table 1). The top SNP is a significant expression quantitative trait locus (eQTL) for *CXCL12* in the kidney glomerulus with the *Pseudomonas*-decreasing allele rs262469-A associated with increased *CXCL12* expression (NephQTL, ref. 20; Supplemental Table 5), while another significant SNP (rs2818912-T, *P* = 2.09 × 10^–8^; *r*^2^ = 0.47 with rs262469-A) is associated with lower *CXCL12* gene expression in testis and ovarian tissue (GTEx v8, ref. 21; Supplemental Table 6). The top SNP in this locus is also a blood eQTL for *ZNF239/MOK2* (eQTLGen, ref. 22; Supplemental Table 7).

Another signal, on chr 3, in an intronic region of *ROBO1*, was associated with class Clostridia relative abundance (lead SNP: rs6802848, *P* = 4.85 × 10^–8^). The Clostridia-decreasing allele, rs6802848-G, is also associated with lower *ROBO1* expression in skeletal muscle (GTEx v8; Supplemental Table 6), and rs1489846-C (*r*^2^ = 0.74 with rs6802848-G) is associated with higher *ROBO1* expression in the kidney glomerulus (NephQTL; Supplemental Table 5).

In the European local genetic ancestry background (Table 1 and Figure 4), we detected an association in an intronic region of *ABCC1* with the class Bacilli and its order Lactobacillales (top SNP: rs246232, *P* = 1.02 × 10^–8^). The top SNP Bacilli- and Lactobacillales-decreasing allele, rs246232-G, is associated with lower *ABCC1* gene expression in blood (eQTLGen; Supplemental Table 7) and is an splicing quantitative trait locus (sQTL) in skeletal muscle, esophagus muscularis, and thyroid for *ABCC1* (Supplemental Table 8). Colocalization analyses between the GWAS loci and eQTL and sQTL reported above did not yield evidence of shared causal variants; therefore, further investigation is required.

Finally, we identified a signal on chr 1, in an intergenic region 196 kb downstream of *BARHL2*, that was associated with family Enterococcaceae relative abundance (top SNP: rs74759570, *P* = 8.84 × 10^–9^). We did not find eQTLs for the top SNPs in this region.

### Phenome-wide association analysis and pleiotropic effects of top signals.

**Figure 5:**
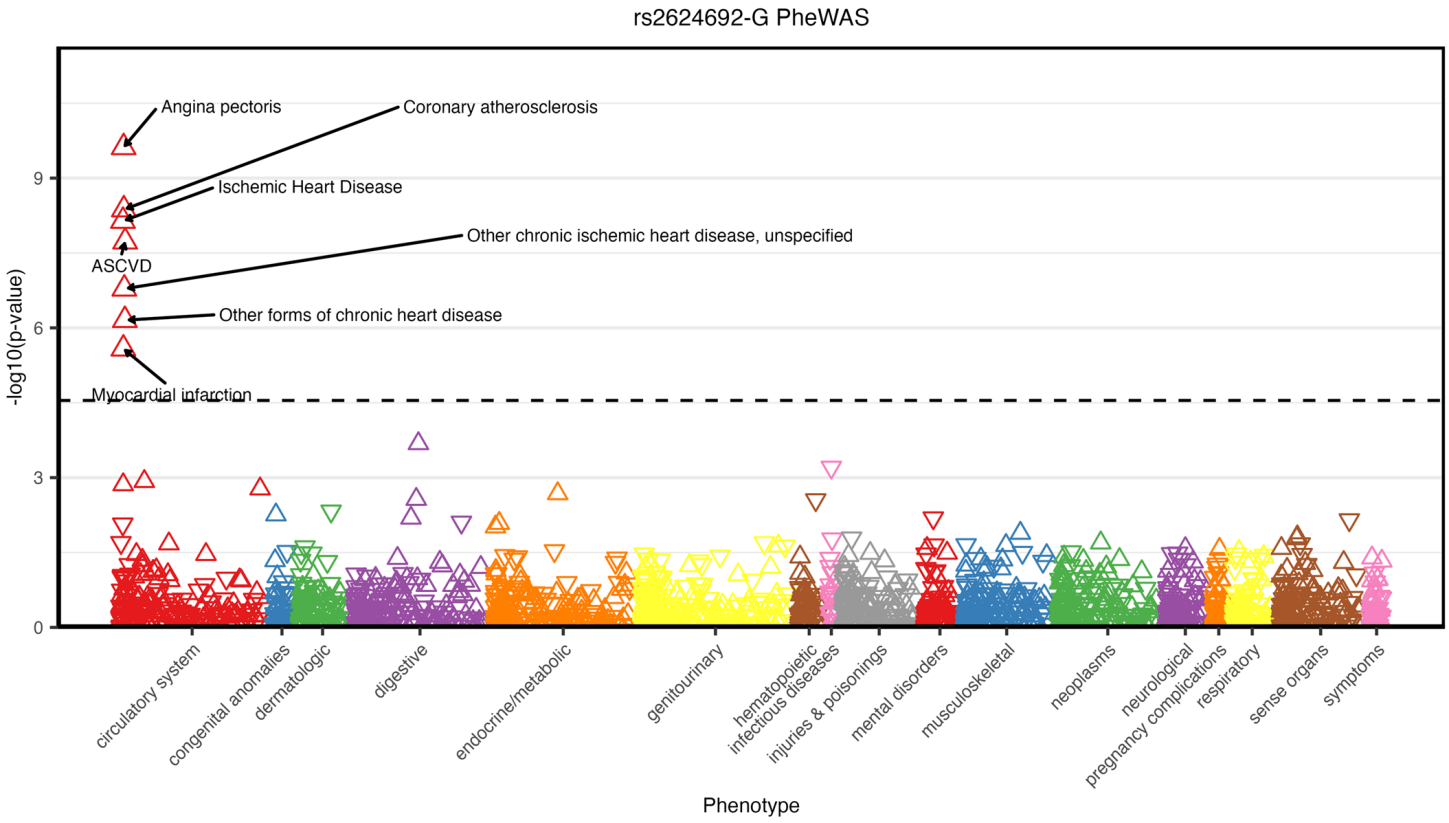
PheWAS for the lead Pseudomonas GWAS SNP. Manhattan plot of phenome-wide association study meta-analysis in the UKBB, eMERGE, and All of Us cohorts for SNP rs2624692. The *x* axis represents phenotypes grouped by categories and the *y* axis the –log(*P* value) of associations. Effect estimates in the PheWAS were calculated with respect to the minor allele G (associated with higher Pseudomonas relative abundance in the GWAS). The dashed line indicates the phenome-wide significance threshold (*P* < 6 × 10^–5^).

**Figure 6:**
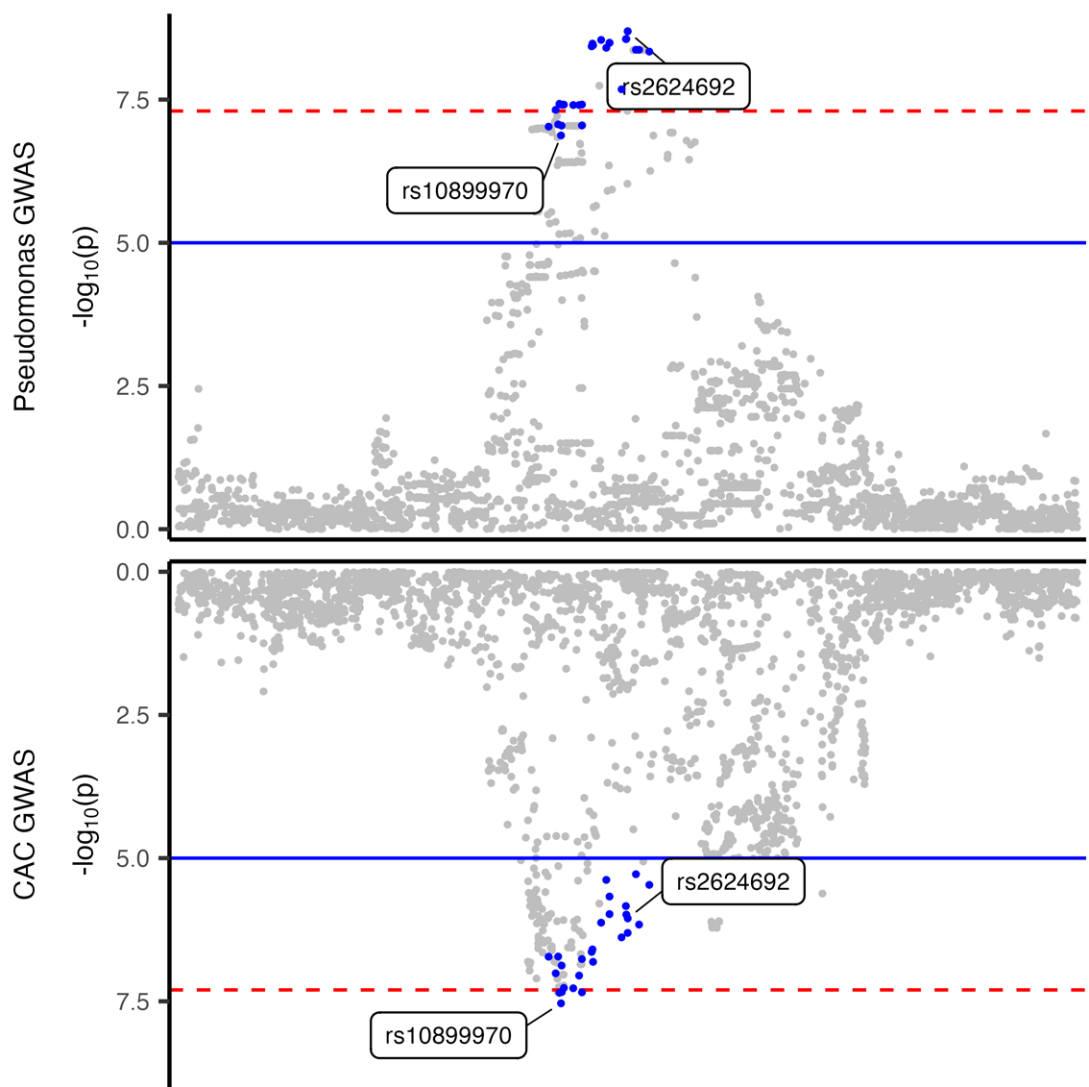
Colocalization of Pseudomonas relative abundance and coronary arterial calcification associations. Regional Miami plot showing the associations with Pseudomonas relative abundance (top panel) and with coronary arterial calcification (bottom panel) in the chr10:43094712-45093012 (hg38) locus (*x* axis) represented as –log(*P* value) on the *y* axis. There is strong evidence of colocalization (PP4 = 0.77). The 95% credible set SNPs are highlighted in blue. Lead SNPs in the signals from each GWAS are labeled with their rs IDs (rs2624692 and rs10899970).

To explore potential effects of the lead SNPs from our bacterial relative abundance GWAS, we carried out a meta–phenome-wide association analysis (meta-PheWAS) across the Electronic Medical Records and Genomics (eMERGE; *N* = 102,138), UK Biobank (UKBB; *N* = 460,358), and All of Us (*N* = 312,944) cohorts combined (Supplemental Tables 9–12). The lead SNP rs2624692 in the *Pseudomonas* GWAS showed significant associations in meta-PheWAS with cardiovascular disease traits (e.g., angina pectoris, *P* = 2.42 × 10^–10^; coronary atherosclerosis, *P* = 4.26 × 10^–9^; Figure 5 and Supplemental Table 9). The association of this locus with coronary heart disease phenotypes is supported by prior studies linking proxy SNPs with coronary artery calcification (CAC) (23), myocardial infarction (24), and coronary artery dominance (25); these studies designated *CXCL12* as the candidate gene. Colocalization analysis showed strong evidence that the *Pseudomonas* relative abundance and CAC associations in these GWAS share a common causal variant (posterior probability = 0.77; Figure 6). Additionally, GWAS catalog matches at this chr 10 locus included the association of a proxy SNP with endometriosis (Supplemental Table 13).

The *ROBO1* signal, associated with *Clostridia* relative abundance, is associated in the PheWAS with diverticulosis and diverticulitis (*P* = 1.09 × 10^–5^) and visual disturbances (*P* = 1.33 × 10^–5^). In addition, the lead SNP in the *ABCC1* locus associated with *Bacilli* relative abundance was associated with testicular cancer (*P* = 2.33 × 10^–5^). This signal is also associated with plasma cysteinyl glycine disulfide levels, a metabolite in the glutathione pathway (26).

### Postinfection induction of Cxcl12 in mouse bladder tissue.

**Figure 7:**
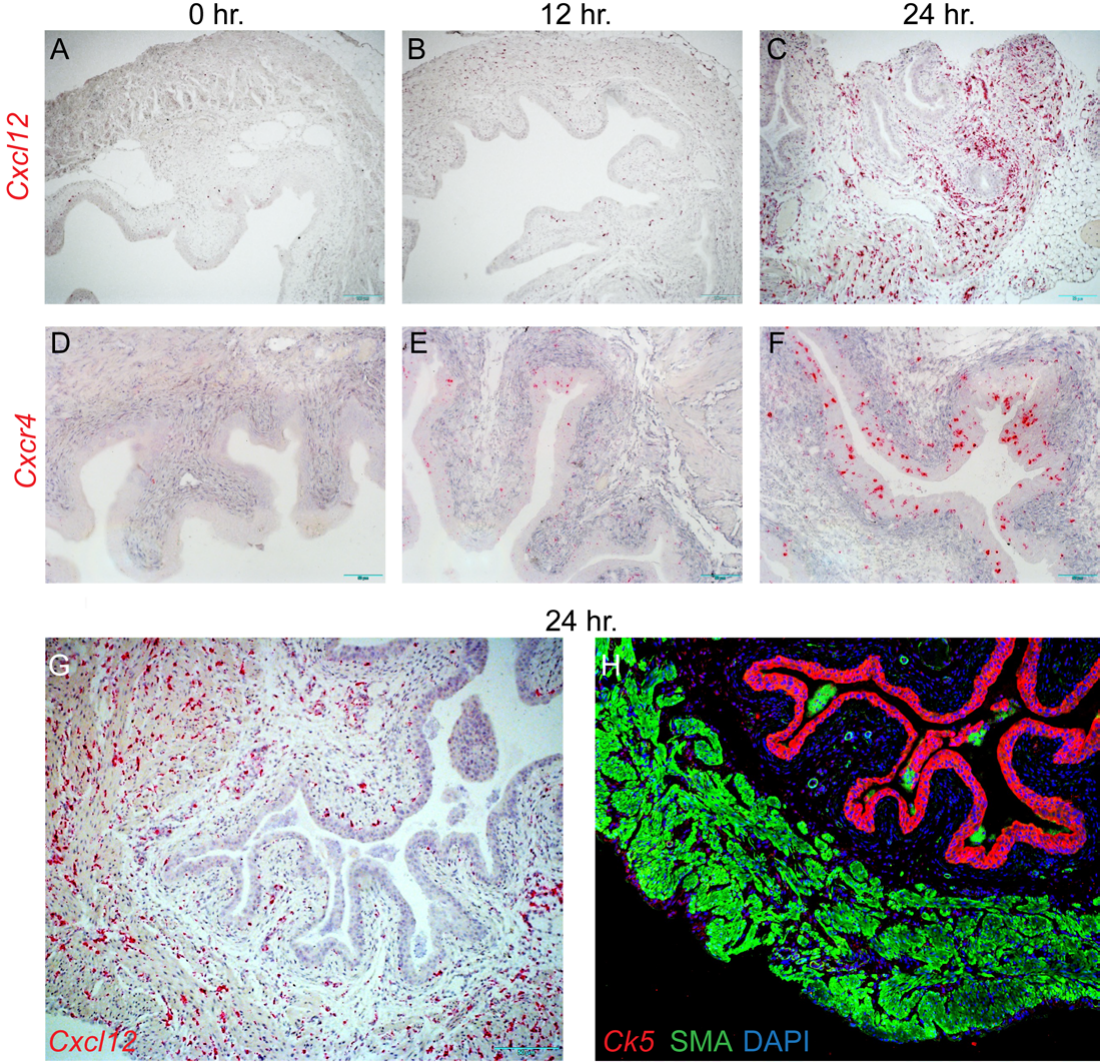
Increased *Cxcl12* and *Cxcr4* expression after UTI in mouse bladder. *Cxcl12* and *Cxcr4* RNA were induced in mouse bladder tissue at 12 hours (**B** and **E**) and more strongly at 24 hours (**C** and **F**) after UPEC infection, but not at 0 hour (**A** and **D**), as seen by chromogenic in situ hybridization (RNAscope). Composite images of consecutive sections from 24 hours postinfection bladder tissue suggest that *Cxcl12* RNA (**G**) localizes mainly to stromal and muscle cell layers, and to a much lesser degree to the urothelium, as marked by smooth muscle actin and cytokeratin 5 immunofluorescence (**H**), respectively. Images shown are representative of 3 mice per time point.

To provide an initial characterization of CXCL12 in the setting of UTI, we induced UTIs with *E*. *coli* strain UTI89GFP (27) in C57BL/6 mice. Neither *Cxcl12* nor its receptor, *Cxcr4*, was expressed at baseline and at 8 hours postinfection. However, we observed induction of *Cxcl12* and *Cxcr4* in mouse bladders 12–24 hours after infection. *Cxcl12* was expressed in smooth muscle layers, where anti–smooth muscle actin immunofluorescence was seen, while *Cxcr4* was expressed in cells in the urothelium, marked by anti–cytokeratin-5 immunofluorescence (Figure 7).

## Discussion

Many comprehensive studies have been conducted on the gut microbiota and its interaction with host genetics in health and disease. However, less is known about the urinary microbiota, specifically in the context of benign urinary tract conditions in children. In this study, we analyzed the urobiota and conducted what we believe is the first pediatric urinary bacterial traits GWAS of patients with UTI and VUR in the RIVUR and CUTIE cohorts. There is evidence suggesting that dysbiosis is an important essential factor in pathogenic bacteria’s invasion of the bladder, particularly in the context of the gut/bladder axis (28, 29). UTI has been associated with a reduction of urobiota α-diversity in young children (30). We found decreased α-diversity with VUR, lack of toilet training, and younger age, suggestive of dysbiosis, immature microbiota, or both. We also detected differences in β-diversity between RIVUR and CUTIE, toilet training status, and age.

The most abundant urinary genera were *Enterococcus*, *Prevotella*, *Pseudomonas*, and *Escherichia/Shigella*. Each of these 4 genera exhibited the highest relative abundance and prevalence in 4 distinct community types, which showed the same associations as measures of bacterial diversity, including with the presence of VUR (RIVUR compared with CUTIE), and with the number of UTIs, and presence of scarring or pyelonephritis, suggesting that susceptibility to recurrent UTIs and pyelonephritis, as well as anatomical defects, might be linked to bacterial composition. While Pseudomonas is infrequently detected by standard urine culture methods, it is not uncommon in the urinary tract when using enhanced culture or sequencing-based approaches (31, 32) and has been associated with the probability of urinary tract anomalies in children with UTI (33). All 4 genera identified — *Escherichia*, *Prevotella*, *Pseudomonas*, and *Enterococcus* — have been associated with urinary symptoms or infections in previous studies. *Escherichia* spp include both commensal strains and those frequently isolated from patients with UTIs, reflecting the context-dependent relationship between bacteria and their host environment; *Prevotella* is a common component of the healthy urobiota, but certain *Prevotella* spp have also been linked to infection (34); and *Enterococcus* spp, while common in humans, include some of the most frequent hospital-acquired pathogens (35). Without comparison with a healthy control group, we cannot rule out that the compositional prevalence of these genera stems from the antibiotic exposure in children treated for UTI.

Our analysis of differential urobiota composition with study variables suggests a more disrupted microbiota in children with altered urine flow (VUR, RIVUR cohort) with an increased relative abundance of taxa that include opportunistic pathogenic and environmental bacteria, such as *Pseudomonas* (4, 36, 37) and *Halomonas* (38, 39), and decreased relative abundance in taxa generally considered beneficial or commensal, such as *Bacteroides*, *Phocaeicola*, *Prevotellaceae*, *Ruminococcaceae*, and *Lachnospiraceae* (40–43). Differential relative abundance profiles observed with age and toilet training are coincident with an increase in α-diversity and possibly reflect the maturation of the urobiota, influenced by anatomical and immunological development with age and changes in the gut-bladder axis with toilet training (44–48). An important caveat when interpreting our results is that the method of urine sample collection was determined by urine toilet training status, which, in turn, correlates with age. Therefore, the associations we observed with bowel and urine toilet training, as well as with age, could reflect different degrees of periurethral contamination with different collection methods.

Our GWAS findings and annotation identify host candidate genes previously implicated in urinary tract development, mechanisms of inflammation and response to infection, and tissue homeostasis. CXCL12, encoded in the chr 10 locus associated with *Pseudomonas* and *Pseudomonadaceae* relative abundances, is a chemokine and natural ligand of CXCR4, and it also binds to CXCR7. It is involved in development, hematopoiesis, immunity, and inflammation (25, 49–51). In the kidney, CXCL12 (aka SDF1) regulates distribution of intercalated cells (α/β-IC). α-IC acidify urine and secrete siderophore lipocalin 2/neutrophil gelatinase-associated lipocalin (LCN2/NGAL) to fight UTI (52, 53). Evidence shows that LCN2 regulates *Cxcl12* expression (54, 55). During development, CXCL12 is involved in ureteric bud branching and mesenchymal tubulogenesis (56). *Cxcl12* has also been implicated in bladder function in normal micturition and inflammation-induced bladder hyperreflexia (57). We demonstrated that in mice, *Cxcl12* and its receptor, *Cxcr4*, are induced at relatively late stages of UPEC infection (12 hours), compared with many cytokines we have previously studied (within 4 hours of infection). Moreover, ligand and receptor are located in distant tissue layers. The apparent localization of *Cxcl12* to the smooth muscle layer of the bladder and *Cxcr4* to the urothelium suggests a role for CXCL12/CXCR4 signaling in the late stages of infection, such as recruiting infiltrating immune cells into the urothelial layer. This aligns with the previously reported role of secreted CXCL12 in attracting immune cells in the mouse bladder after infection (58). Alternatively, this signaling pathway might be involved in regeneration following infection. There are other examples from bladder physiology that are consistent with intertissue signaling, such as urothelial basal cell SHH signaling leading to increased secretion of WNT proteins by stromal cells during injury and regeneration (59).

Our pheWAS of the top SNP in the *CXCL12* locus detected the known association of *CXCL12* with cardiovascular phenotypes, and follow-up colocalization analysis suggested the same causal variant for the association with increased *Pseudomonas* relative abundance and CAC. Coincidentally, VUR has been linked to subclinical cardiovascular disease in children, even in the absence of renal scarring (60). We can hypothesize that chronic inflammation resulting from UTI may lead to an increased risk of cardiovascular disease later in life and that common pathways involving CXCL12/CXCR4/CXCR7 between vascular and bladder/vesicoureteral junction development and remodeling cannot be ruled out either. A recent study (25) has shown that Cxcl2 haploinsufficiency is associated with alterations in cardiac vasculature in mice, which may explain the association with cardiovascular traits. One can therefore also hypothesize that *CXCL12* variation may also predispose to structural abnormalities in the urinary tract and result in altered interactions with the urinary microbiome.

Regarding other risk loci, biallelic pathogenic variants in *ROBO1*, associated in this study with *Clostridia* relative abundance, have been linked to syndromic CAKUT, including VUR, while *Robo1^Ile270Thr/Ile270Thr^* mice have renal anomalies and hydroureter (61). Prior studies have shown that SLIT2/ROBO1 signaling influences macrophages and neutrophil function and can affect susceptibility to bacterial infection (62–64). An association with *Clostridia* infection has not been specifically reported. MRP1, the product of *ABCC1*, associated with *Bacilli* and *Lactobacillales* relative abundances in our study, negatively regulates intracellular glutathione abundance in human cells, increasing ferroptosis sensitivity (65). Cross-referencing of this signal with the GWAS catalog also points to a possible link with the glutathione pathway. Ferroptosis, which entails iron accumulation and lipid peroxidation, is involved in response to infection and bladder cancer (66–69). The *Enterococcaceae* relative abundance association is intriguing since this family includes the genus *Enterococcus*, with species known to cause catheter-associated UTIs (70, 71). However, no clear candidate gene arose from our analysis of this intergenic locus.

Our study has several limitations, including the incomplete resolution and scope of 16S rRNA gene sequencing to identify bacterial traits compared with metagenomic analysis; the relatively modest sample size, especially for detecting genetic associations in a multiethnic cohort; the unbalanced sex distribution of our study cohort; the lack of a healthy population control sample; and the heterogeneity in infection history, antibiotic treatment, and inflammation intrinsic to the patient population that our samples are drawn from. Extension and replication of our findings with larger cohorts are needed. Additionally, further functional studies are warranted based on our results, such as colocalization with eQTL in human bladder tissue and further animal studies to clarify the involvement and role of candidate genes.

Our results, compared with a recent study in a healthy young adult East Asian population sample (72), show marked differences in bacterial composition and its association with genetic factors. These differences potentially reflect changes in the urinary microbiome with UTI and VUR, age, sex, disease state, possibly demographic and geographic factors, and genetic background.

Our study is the first to our knowledge to analyze the microbiota and its host-genetic associations in pediatric UTI and VUR. We characterized the diversity and relative abundance profile and identified 4 community types in the urobiota of this patient population sample and showed their associations with study variables. We also identified 4 genetic loci, and the candidate genes *CXCL12*, *ABCC1*, and *ROBO1*, associated with urinary bacterial traits. Furthermore, our results suggest a possible link between pediatric UTI and cardiovascular disease later in life, mediated by CXCL12.

## Methods

### Sex as a biological variable.

Our study examined data from both male and female patients. It was limited to patients from the RIVUR and CUTIE cohorts; therefore, it involved significantly more female (91%) than male participants (Supplemental Tables 1 and 4). Mice in our study were female, as this better matched our human study.

### Participants and specimens.

We utilized urine and genomic DNA samples from participants in the RIVUR study (children with documented VUR, recruited after a first or second UTI) and the CUTIE (children with UTI, and without VUR), a companion study to RIVUR.

In the RIVUR and CUTIE studies, urine specimens were collected by means of catheterization or suprapubic aspiration from children who were not toilet-trained and by the clean-voiding method from toilet-trained children (2, 3); urine samples were kept unfrozen before shipment to the NIDDK biorepository, shipped on ice packs within the hour, or refrigerated until shipment. Frozen samples received from the repository were thawed on ice in our laboratory immediately before processing. RIVUR samples were obtained before randomization.

Anonymized genomic DNA (*N* RIVUR = 456, *N* CUTIE = 163), frozen urine (–80°C; *N* RIVUR = 276, *N* CUTIE = 101), and phenotypic data were obtained from the NIDDK Central Repository (NIDDK-CR).

### Urinary microbiota 16S rRNA gene sequencing and data processing.

The 16S rRNA gene V3-V4 region from 375 urine samples was amplified using standard primers (73), then sequenced on an Illumina MiSeq system using the MiSeq Reagent Kit v3 (2 × 300 bp), following contamination-control procedures described for low-biomass microbiome studies (74, 75).

FASTQ files were processed using the R package dada2. Primers’ sequences were removed with Cutadapt software (76). Sequences were truncated to 240 bp of the forward and 200 bp of the reverse strand. Reads with a quality score < 5, or with errors > 2 in the forward or > 5 in the reverse strand, were discarded. Error rates were learned for the forward and reverse reads, and all identical sequencing reads were combined into “unique sequences.” Sequence variants were inferred using the dada algorithm, and the forward and reverse reads were merged. A table of amplicon sequence variants (ASVs) was constructed of merged reads, and chimeric sequences were removed. Taxonomy was assigned with the Ribosomal Database Project (RDP) Naive Bayesian Classifier algorithm implemented in the package, using the RDP trainset 18 (dp_train_set_18.fa.gz) (77). Further processing was done using the phyloseq R package (78). Only samples with at least 5,000 total reads were carried forward, resulting in a set of 325 samples (*n* RIVUR = 228, *n* CUTIE = 97). Children in this combined cohort (*N* = 325) had a median age of 24 months, and 92% were female (Supplemental Table 1). A subset of these (*N* = 278, *n* RIVUR = 193, *n* CUTIE = 85) also had host genomic DNA genotyping high-quality data (see below). For both sets (*N* = 325 and *N* = 278), ASVs with abundances of less than 2 counts across samples were removed.

### Analysis of α- and β-diversity and community types.

Abundances per sample were normalized by resampling with replacement to total counts equal to the sample with the fewest total counts using the rarefy_even_depth phyloseq function. Computation of per-sample Shannon α-diversity was done with the estimate_richness function; rank-based regression with the Rfit R package function rfit was used to test for associations of α-diversity with study variables; and for the cohort variable, race and ethnicity were included as covariates to correct for differences in these demographic variables between RIVUR and CUTIE in our dataset. Pairwise β-diversity was computed with the phyloseq distance function (Bray method), and subsequent principal coordinate analysis of the resulting distance matrix was performed using the ordinate function. Permutational analysis of variance (PERMANOVA) on Bray dissimilarity was performed with the vegan R package adonis function (permutational MANOVA) (79).

JSD distance metrics were calculated for relative genera abundances followed by partitioning around medoids (PAM) clustering using the R packages phylentropy and cluster. The optimal number of clusters was determined by the silhouette method with the factoextra R package function fviz_nbclust. Linear discriminant analysis effect size (LEfSe implemented in the R package microbiomeMarker) was used to determine the genera dominating each cluster. To estimate associations between continuous or ordinal variables and cluster membership, the Kruskal-Wallis was used, followed by Dunn’s test; the Fisher’s exact test was used for categorical variables.

### Core microbiome and differential abundance analysis.

Using the R package phyloseq, reads per taxon per sample at each taxonomic rank level obtained with the tax_glom function were normalized with the rarify_even_depth function. Next, only taxa with at least 5 reads in at least 10% of samples were retained. For differential abundance analysis at each taxonomic level, a consensus approach (80) between 2 methods was used: analysis of compositions of microbiomes with bias correction (ANCOM-BC2) (15) and microbiome multivariable association with general linear models (MaAsLin2) (16).

### Genotyping and imputation.

Genomic DNA samples from RIVUR (*N* = 456) were genotyped on Illumina MEGA1.0 arrays and samples from CUTIE (*N* = 163) on Illumina MEGAeX arrays. Genotyping calls were generated in Illumina GenomeStudio v2, exported, and further processed with PLINK1.9. Samples from RIVUR and CUTIE were merged into one genotyping dataset comprising 1,981,066 unique biallelic SNPs. Individuals with per-sample call rate < 90% in high-quality common SNPs, with discordant genotype estimated/reported sex checks, or without corresponding urine samples were excluded. The remaining 320 genotyped samples were retained for further analysis. Kinship analysis with the KING software identified 3 full sibling pairs. Ancestry inference was also conducted using KING with 1000 Genomes reference data for projection on 5 genetic ancestry groups: Africans (AFR), Admixed Americans (AMR), East Asians (EAS), Europeans (EUR), and South Asians (SAS) (Supplemental Figure 7). Prior to imputation, further filters were applied separately to the AFR, EUR, and combined AMR-EAS-SAS, excluding SNP missingness rate > 5%, minor allele frequency (MAF) > 0.1%, and deviations from Hardy-Weinberg equilibrium *P* < 1 × 10^–4^, and updating or removing SNPs with discrepancies in strand, alleles, and position based on a 1000 Genomes Project reference, using PLINK software and the HRC-1000G-check-bim-v4.3.0 perl script from the McCarthy Group.

The TOPMed Imputation Server was used to carry out phasing (EAGLE v2.4) and imputation (MINIMAC 4) with the TOPMed r2 reference panel (hg38). Samples from all ancestries were imputed together, resulting in a common set of postimputation SNPs. ChrX was imputed separately for males and females. The HLA region was extracted with bcftools and imputed separately using the Michigan Imputation Server with the multiethnic HLA reference panel (81) (hg19; chr6:28,000,361–33,966,845). Server parameters were set to Four-digit Multi-ethnic HLA reference panel (GRCh37/hg19), with phasing with EAGLE v2.4, standard quality control, and imputation. The imputed HLA genotypes were converted to hg38 with Picard LifoverVcf. Following imputation, only biallelic SNPs with imputation *r*^2^ ≥ 0.8 and Hardy-Weinberg test *P* ≥ 1 × 10^–15^ were carried forward in the analysis.

### Genome-wide association analyses.

GWAS were performed on 278 samples (193 RIVUR and 85 CUTIE participants, median age of 24 months, 91% female; Supplemental Table 2), corresponding to the intersection of those that passed both host genomic DNA genotyping (*N* = 320) and urinary bacterial 16S rRNA gene sequencing (*N* = 325) quality controls and filters described above.

Relative abundances in the core microbiome were computed for each sample.

Associations of core microbiota taxa nonzero relative abundances with host genome-wide genotyped and imputed SNPs were performed using 2 approaches. First, using REGENIE (18) software, with subcohort (RIVUR or CUTIE), sex, age (in months) at baseline, clinic site, toilet training status for bladder and bowel at baseline (TTUB), antibiotic treatment at baseline before sample collection and before randomization in the RIVUR study (AB), and 10 genetic principal components (PCs) as covariates. PCs were computed with PCAiR software (Supplemental Figure 8) based on data from genotyped autosomal SNPs LD-pruned with PLINK1.9. Phenotypes were normalized using rank-based inverse normal transformation (RINT), and a minor allele count > 20 filter was applied. REGENIE Step1 involved fitting a ridge regression model using 520,842 genotyped SNPs. REGENIE Step2 used the predictions from Step1 to perform genome-wide association testing using 6,148,017 high-quality postimputation SNPs. GWAS results were visualized using Manhattan, quantile-quantile, and LocusZoom (82) plots. Second, given the multiancestry nature and small size of our study cohort, we used a local genetic ancestry-aware approach implementing the TRACTOR framework (83), on 275 unrelated samples. Local ancestry inference was performed on postimputation genotype data with RFMix v2, after phasing with SHAPEIT4. The phased AFR, EUR, and EAS genetic ancestry groups from the 1000 Genomes Project (Phase 3; *N* = 2,504; hg38) were used as reference. TRACTOR was then used to extract per-sample ancestry-specific haplotype tract counts and SNP dosages, followed by fixed-effect model linear regression using R, with subcohort (RIVUR/CUTIE), sex, age (in months), TTUB, AB, clinic site, haplotype counts, and RFMix-computed admixture fractions as covariates. A local ancestry MAF > 0.05 filter was applied. To assess the possible presence of additional independent genome-wide significant SNPs within GWAS loci, conditional analyses were conducted with both REGENIE and TRACTOR conditioning for the genotype of the lead SNP in each signal, and LD-based clumping was also performed with PLINK1.9 and 1000 Genomes Project Phase 3 genome build hg38 for all populations (1000G ALL) and European genetic ancestry populations (1000G EUR) reference panels.

### Functional annotation and phenome-wide association studies.

For each GWAS significant signal, all SNPs within ±500 kb of the lead SNP were first selected if they were in at least moderately strong linkage disequilibrium (*r*^2^ ≤ 0.5, based on the 1000G EUR reference panel) with the lead SNP or were genome-wide significant in our GWAS. These SNPs were then annotated with VEP software and cross-referenced with the GWAS catalog (84); eQTL from GTEx (21) v8, NephQTL (85), eQTLGen (22), DICE (86), meQTL from Liu et al. (87); sQTL from GTEx v8; blood pQTL (88); metabolomic GWAS (26); and microbiota and microbiome GWAS (17, 72, 89–95). Positive cross-reference matches were tabulated in the supplement and/or cited in the main text. CAC GWAS (23) summary statistics were obtained from the EMBL-EBI GWAS catalog (accession number: GCST90278455). Colocalization analysis was conducted with the R Coloc package, taking a PP4 > 0.7 as strong colocalization evidence.

PheWAS for lead genome-wide significant SNPs was performed as previously described (96, 97) using the PheWAS R package, on data from 3 biobanks: the Electronic Medical Records and Genomics III (eMERGE-III) (96, 98), All of Us (99), and the UK Biobank (100). Meta-PheWAS statistics were then calculated by meta-analysis of PheWAS results across the 3 biobanks. Associations with *P* < 0.05/(number of tested phenotypes) were considered statistically phenome-wide significant.

### Animal studies.

Two-month-old female C57BL/6 mice (*n* = 3 per time point) were infected with the *E*. *coli* UTI89GFP strain, an isogenic derivative of UTI89 (27) (50 μL 10^8^ CFU/mL) from a clinical isolate via a soft polyethylene catheter (PE10 tubing: 0.011 in. internal; 0.024 in. external diameter) without external pressure (52). In situ hybridization was performed on mouse bladder tissue FFPE sections using the chromogenic RNAscope 2.5 HD reagent kit (RED, ACD, catalog 322350) and the RNAscope 2.5 HD duplex reagent kit (ACD, catalog 322430) according to the manufacturer’s protocols. Both *Cxcr4* and *Cxcl12* C2 probes were applied at 1/25 dilution in RNAscope Diluent. For immunofluorescence staining, FFPE sections were deparaffinized in xylene and hydrated via alcohol gradient. Slides were boiled in antigen retrieval solution (from RNAscope) at 95°C for 15 minutes and blocked in 3% BSA in 0.125% Triton in PBS for 1 hour. Primary antibodies (Mouse anti–smooth muscle actin, Santa Cruz Biotechnology catalog sc-32251, at 1/100 dilution; Chicken anti–cytokeratin-5, BioLegend, at 1/500 dilution) were applied overnight at 4°C in blocking buffer. Slides were washed 3 times in PBS and then incubated in secondary fluorescent antibodies (Donkey anti-Chicken Alexa Fluor 647, catalog 703-605-155, and Goat anti-Mouse Alexa Fluor 594, catalog 115-585-003, Jackson ImmunoResearch) for 1 hour at room temperature. Slides were washed 6 times in PBS and imaged with a ZEISS LSM 710 confocal microscope.

### Statistics.

Associations of α-diversity with study variables was conducted with rank-based regression. Pairwise β-diversity was computed with the Bray method, and PERMANOVA on Bray dissimilarity was performed subsequently. For clustering of samples based on their genera composition, JSD distance metrics were calculated, followed by PAM clustering. To estimate associations between continuous or ordinal variables and cluster membership the Kruskal-Wallis and Dunn tests were used and Fisher’s exact test for categorical variables. Analysis of compositions of microbiomes with bias correction (ANCOM-BC2) (15) and microbiome multivariable association with general linear models (MaAsLin2) (16) were used for differential bacterial relative abundance analysis. All microbiota statistical analyses were conducted in R. GWAS were performed on RINT normalized traits with 2 statistical methods: a mixed effects regression model implemented in REGENIE (18) software and a linear regression model within the TRACTOR framework (83). PheWAS was conducted using logistic regression, followed by inverse variance meta-analysis as implemented in the PheWAS R package. Associations with *P* < 0.05/(number of tested phenotypes) were considered statistically phenome-wide significant.

### Study approval.

Use of human specimens and data provided by the NIDDK-CR under material and data use agreements was approved by the Columbia University Institutional Review Board. Mouse husbandry, infection, and euthanasia followed protocols approved by the Columbia Institutional Animal Care and Use Committee.

### Data availability.

Host genomic DNA genotyping data have been submitted to dbGaP under accession number phs001749 and can be accessed through authorized access. Bacterial 16S rRNA sequences are available from the NCBI Sequence Read Archive under accession number PRJNA1330300. Phenotypic data, including clinical and demographic variables, are available from the NIDDK-CR (https://repository.niddk.nih.gov/study/51). Only publicly available open-source software and R (v4.2.2) and Python (v3.8.10) packages and workflows were used: PLINK v1.9 (https://www.cog-genomics.org/plink/1.9), SHAPEIT v4.2, RFMix v2.03 (https://github.com/slowkoni/rfmix), REGENIE v2.2.4 (https://github.com/rgcgithub/regenie), TRACTOR (https://github.com/Atkinson-Lab/TRACTOR), dada2 v1.24.0 (https://github.com/benjjneb/dada2), phyloseq v1.40.0 (https://github.com/joey711/phyloseq), ANCOM-BC2 v1.6.4 (https://github.com/FrederickHuangLin/ANCOMBC), MaAsLin2 v1.10.0 (https://github.com/bioconductor-source/MaAsLin2), PheWAS v0.99.6.1 (https://github.com/PheWAS/PheWAS), and coloc v5.2.3 (https://cran.r-project.org/web/packages/coloc). The specific functions and parameters used are described in the Methods subsections above. No software packages or custom analysis algorithms were developed for this study. The blood eQTL by eQTLGen is available at https://www.eqtlgen.org/; NephQTL is available at http://nephqtl.org/; GTEx is available at https://gtexportal.org/home/; GWAS catalog is available at https://www.ebi.ac.uk/gwas Values in plots are reported in the Supporting Data Values file.

## Author contributions

MV, ACU, JB, and AGG conceptualized the study. MV, P Khosla, DB, AK, YG, HP, THS, AG, KX, IAG, P Krithivasan, JM, TS, and TYL contributed to the investigations and formal analyses (experiments, computational and statistical analyses, and visualizations). VK contributed to the project administration. MV, P Khosla, DB, AK, YG, HP, P Krithivasan, TS, and TYL contributed to data curation. AK, NAL, YL, HH, SSC, KK, and ACU contributed resources. MV, AG, JB, and AGG contributed to the writing by preparing the original draft. P Khosla, DB, AK, HK, THS, KK, and ACU contributed to the writing by editing the original draft. All authors reviewed the manuscript. CLM, ACU, JB, and AG contributed to funding acquisition. AGG supervised the study.

## Funding support

This work is the result of NIH funding, in whole or in part, and is subject to the NIH Public Access Policy. Through acceptance of this federal funding, the NIH has been given a right to make the work publicly available in PubMed Central.

- Columbia University O’Brien Center for Benign Urology (NIH NIDDK grant U54 DK104309).
- eMERGE Network by the NIH National Human Genome Research Institute through grant 5U01-HG008680.
- All of Us Research Program by the NIH Office of the Director through the following grants: Regional Medical Centers: 1OT2OD026549; 1OT2OD026554; 1OT2OD026557; 1OT2OD026556; 1OT2OD026550; 1OT2OD 026552; 1OT2OD026553; 1OT2OD026548; 1OT2OD026551; 1OT2OD026555; IAA# AOD16037; Federally Qualified Health Centers: HHSN 263201600085U; Data and Research Center: 5U2COD023196; Biobank: 1U24OD023121; The Participant Center: U24OD023176; Participant Technology Systems Center: 1U24OD023163; Communications and Engagement: 3OT2OD023205; 3OT2OD023206; and Community Partners: 1OT2OD025277; 3OT2OD025315; 1OT2OD025337; 1OT2OD02527.

## Supplementary Material

Supplemental data

Supplemental tables 1-4

Supplemental tables 5-13

Supporting data values

## Figures and Tables

**Table 1:**
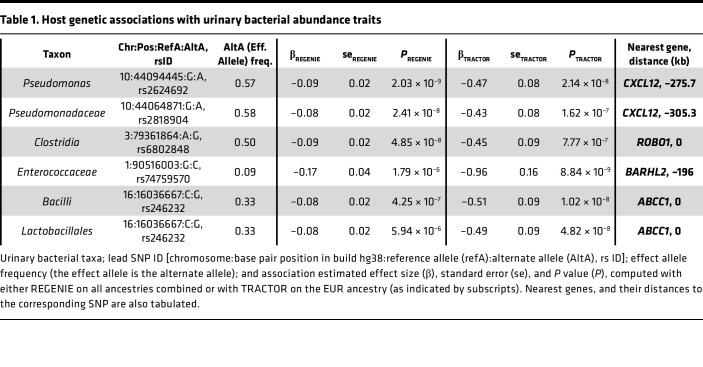
Host genetic associations with urinary bacterial abundance traits

## Version 1 12/09/2025

In-Press Preview

## Version 2 01/23/2026

Electronic publication
