# Supplementary material for: Urobiota analysis and genome-wide association study in pediatric recurrent urinary tract infections and vesicoureteral reflux
Source: JCI Insight. 2025 Dec 9;11(2):e199689. doi: 10.1172/jci.insight.199689 (PMC12892914; doi:10.1172/jci.insight.199689)
Supplement: Supplemental data [file jciinsight-11-199689-s027.pdf]

Supplemental Note: Tests for differences in dispersion in α-diversity and β-diversity

We tested for differences in dispersion in α-diversity between groups (using the Fligner-Killeen test of homogeneity of variance) and with continuous variables, such as age, (using the Breusch–Pagan test), as well as for differences in dispersion in β-diversity between groups (using the analysis of multivariate homogeneity of group dispersions implemented in the R vegan package function betadisp, followed by ANOVA on its results) and with age (by applying the Breusch–Pagan test to the distance to the centroid regressed on the continuous variable). We did not find evidence of statistically significant differences in dispersion ($P > 0.05$) with any of the variables shown in Figure 1 and Supplemental Figure 1, indicating that the statistically significant differences we report for α- and β-diversity are based on differences in measures of centrality.

Supplemental Figures

Supplemental Figure 1. Urobiota α- and β-diversity associations with blood pressure.

Supplemental Figure 2. Urobiota composition heatmaps.

Supplemental Figure 3. Bacterial community types. Cluster plots and abundance and prevalence of top genera per cluster.

Supplemental Figure 4. Differential bacterial taxa abundance with bowel and urine toilet training.

Supplemental Figure 5. Differential bacterial taxa abundance with age.

Supplemental Figure 6. Phylogenetic tree of 62 taxa present in at least 50% of 278 urine samples in the GWAS.

Supplemental Figure 7. Inferred ancestry.

Supplemental Figure 8. Principal component analysis.

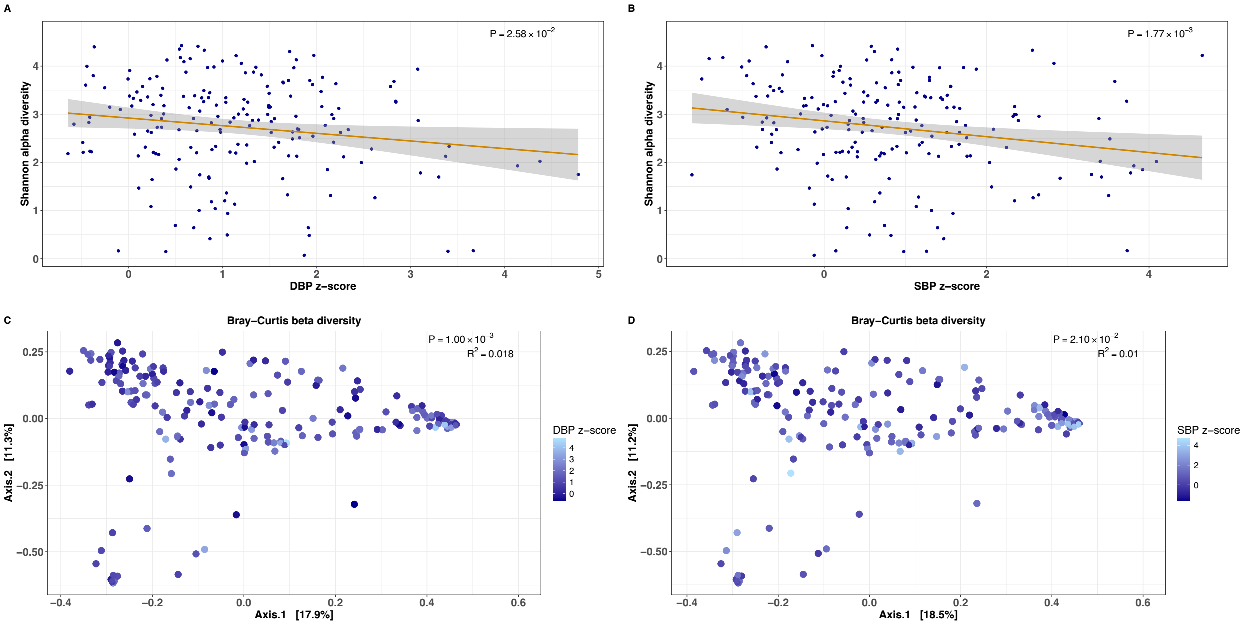

Supplemental Figure 1. Urobiota α- and β-diversity associations with blood pressure. Shannon α-diversity **(top)** and Bray-Curtis β-diversity **(bottom)** were computed and tested for differences with diastolic blood pressuer (**A, C**; DBP; z-score based on height, sex, and age) and systolic blood pressure (**B, D**; SBP; z-score based on height, sex, and age).

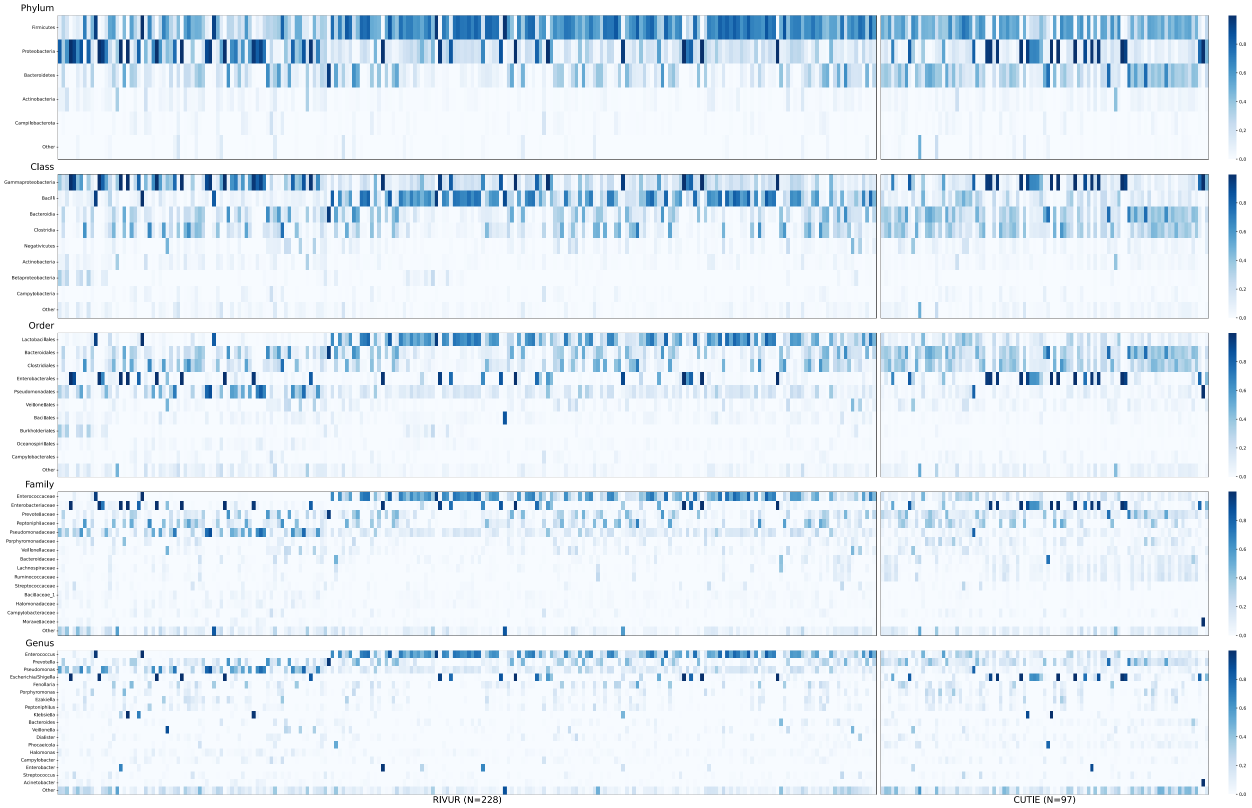

Supplemental Figure 2. Urobiota composition. Heatmap showing bacterial relative abundances (darker shade of blue represents higher relative abundance) at the Phylum, Order, Class, Order, Family, and Genus taxonomic levels (panels from top to bottom) in the RIVUR (left panels) and CUTIE (right panels). Each column represents one urine sample; rows represent taxa. Taxa with relative abundance < 0.1% are collapsed under “Other” at each taxonomic level.

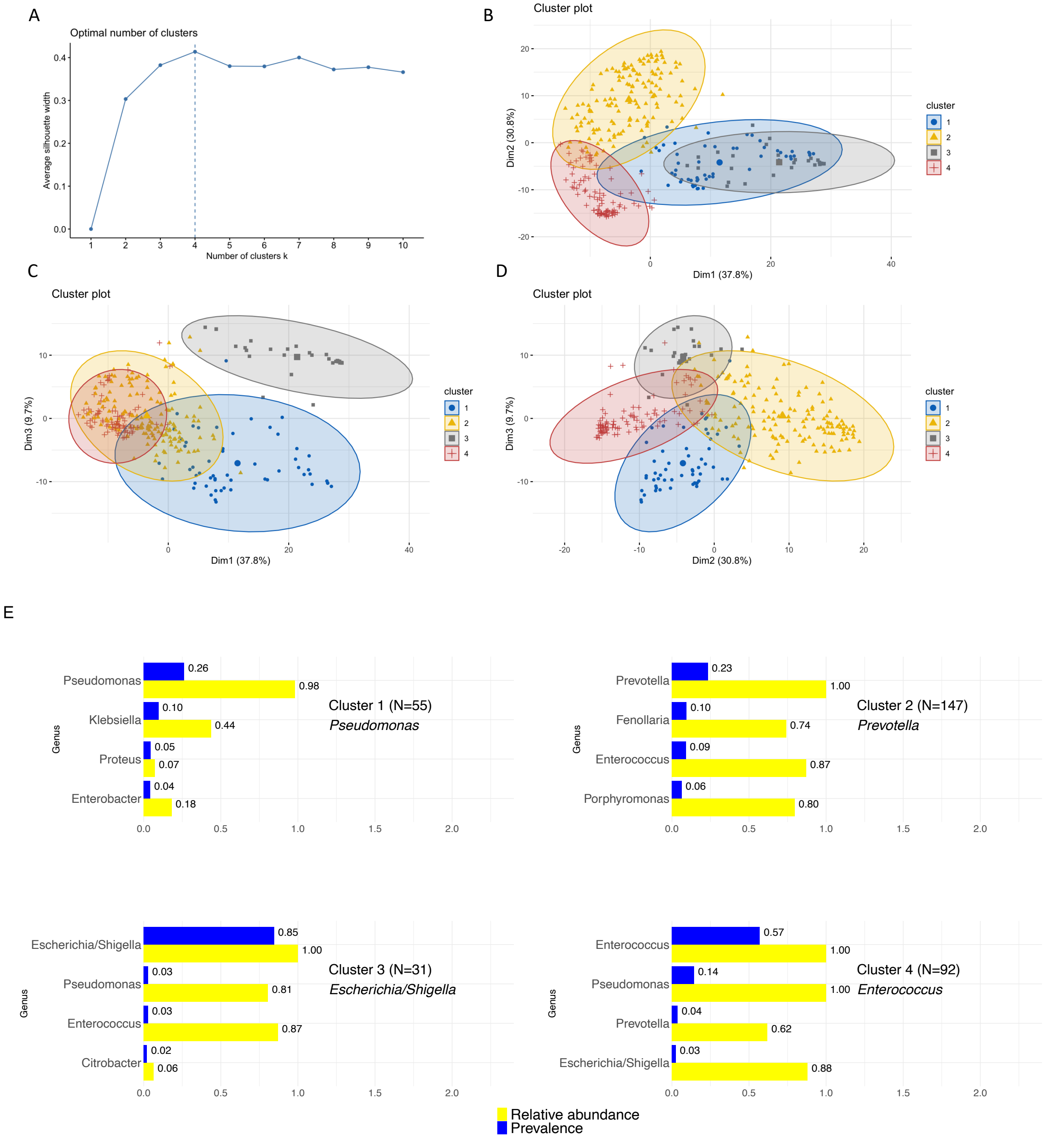

Supplemental Figure 3. Bacterial community types. The optimal number of clusters was determined by the Silhouette method (A). Cluster plots (PCA) the distribution of urine samples in four clusters based on the relative abundances of genera (B-D; 3-dimensional data is presented as three 2-dimensional plots). Aggregate relative abundance (blue bars) and prevalence (yellow bars) of top genera are plotted for each cluster (E).

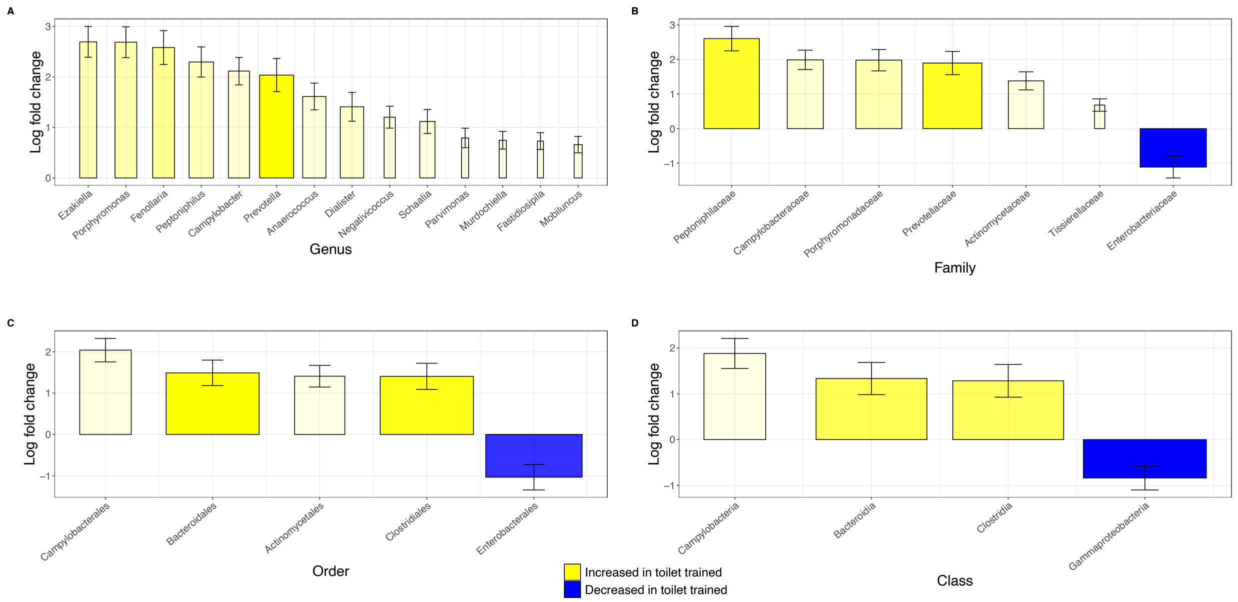

Supplemental Figure 4. Differential bacterial taxa abundance with bowel and urine toilet training. Bar plots represent analysis of compositions of microbiomes with bias correction (ANCOM-BC2) at the Genus (A), Family (B), Order (C), and Class (D) taxonomic levels. Increased abundance with toilet training is represented in yellow, and decreased abundance in blue. Color intensity is proportional to total relative abundance and bar width to prevalence across both cohorts. Only statistically significant ($q < 0.05$) differences that could also be detected with MaAsLin2 are shown.

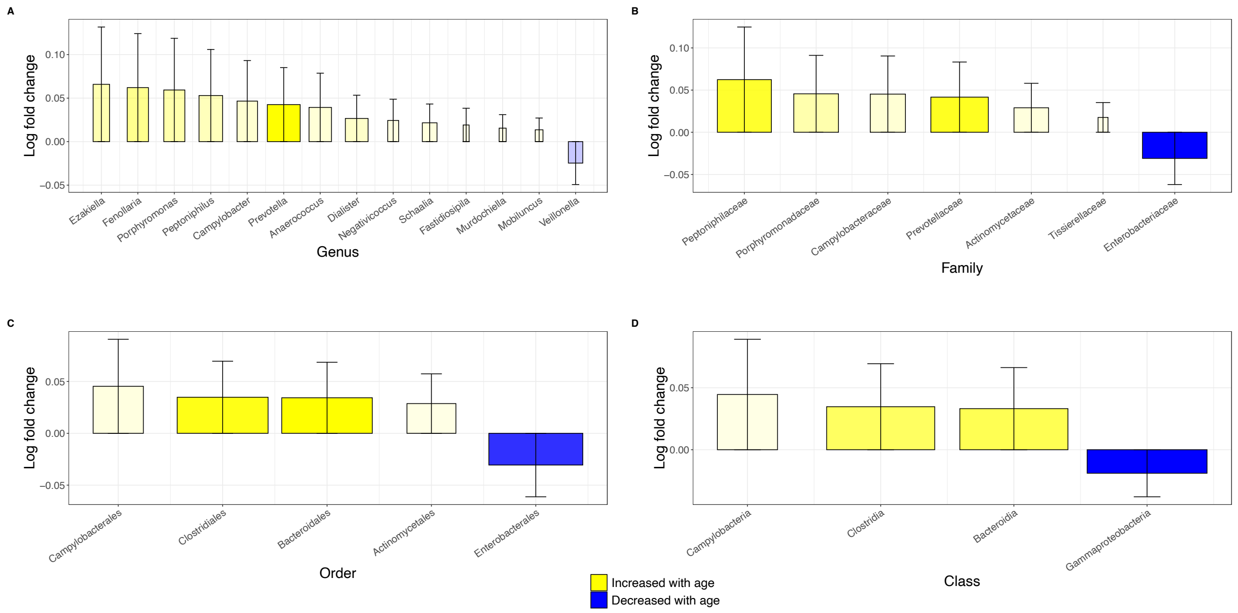

Supplemental Figure 5. Differential bacterial taxa abundance with age. Barplots represent analysis of compositions of microbiomes with bias correction (ANCOM-BC2) at the Genus (A), Family (B), Order (C), and Class (D) taxonomic levels. Increased abundance with age is represented in yellow, while decreased abundance is represented in blue. Color intensity is proportional to total relative abundance and bar width to prevalence across both cohorts. Only

statistically significant ($q < 0.05$) differences associated with age (adjusted for cohort and sex) that could also be detected with MaAsLin2 are shown.

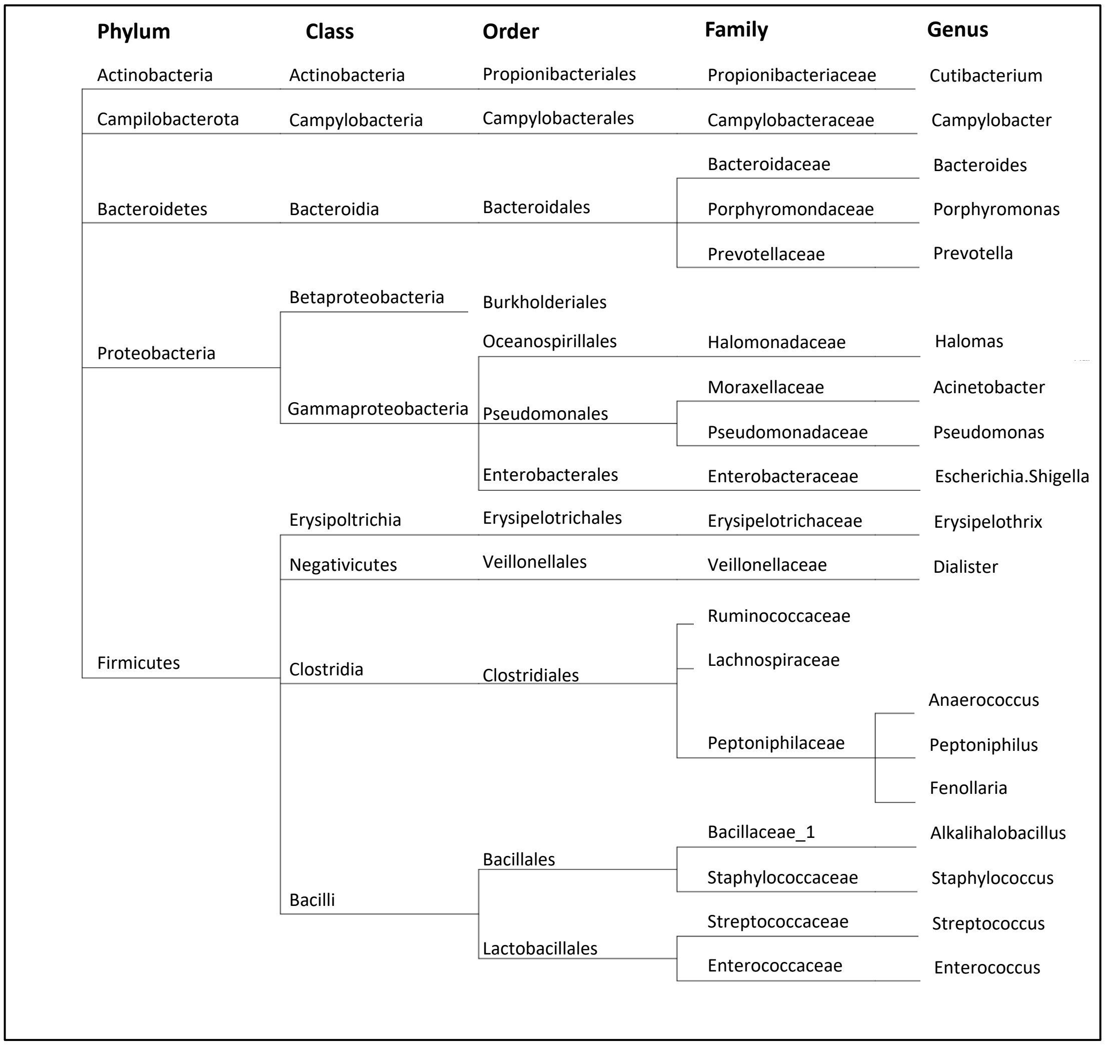

Supplemental Figure 6. Phylogenetic tree of 62 taxa present in at least 50% of 278 urine samples in the GWAS.

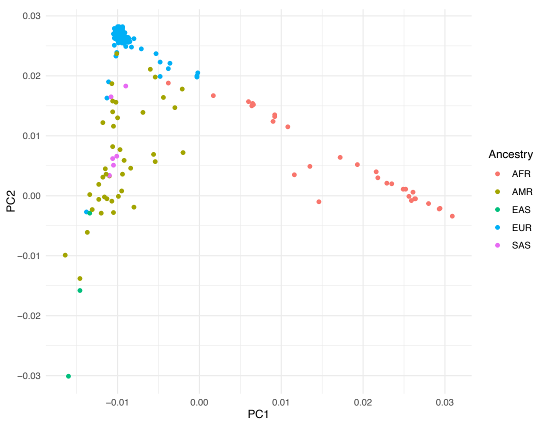

Supplemental Figure 7. Inferred ancestry. Principal components plot from initial King software ancestry inference for 320 genotyped RIVUR and CUTIE participants' samples passing genomic DNA genotyping quality control (see methods). 1000 Genomes reference data was used for projection on five genetic ancestry groups: Africans (AFR), Admixed Americans (AMR), East Asians (EAS), Europeans (EUR), and South Asians (SAS).

-

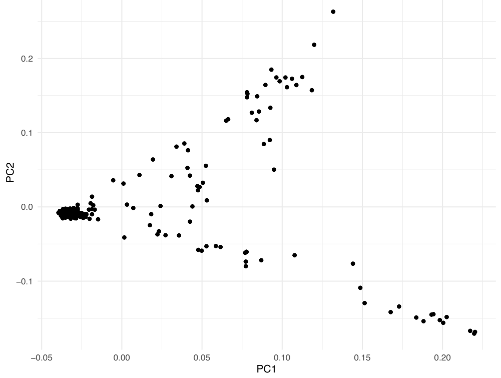

Supplemental Figure 8. Principal component analysis. Plot of principal components computed with PC-AiR for 278 RIVUR and CUTIE participants passing genomic DNA genotyping and urine 16S RNA gene sequencing quality control and filtering (see methods).

-
